# Supplementary material for: Osteopontin, Macrophage Migration Inhibitory Factor and Anti-Interleukin-8 Autoantibodies Complement CA125 for Detection of Early Stage Ovarian Cancer
Source: Cancers (Basel). 2019 Apr 28;11(5):596. doi: 10.3390/cancers11050596 (PMC6562667; doi:10.3390/cancers11050596)
Supplement: Supplementary file 1 [file cancers-11-00596-s001.pdf]

Supplementary Materials: Osteopontin, Macrophage Migration Inhibitory Factor and Anti-Interleukin-8 Autoantibodies Complement CA125 for Detection of Early Stage Ovarian Cancer

Jing Guo, Wei-Lei Yang, Daewoo Pak, Joseph Celestino, Karen H. Lu, Jing Ning, Anna E. Lokshin, Zhongping Cheng, Zhen Lu and Robert C. Bast, Jr.

A

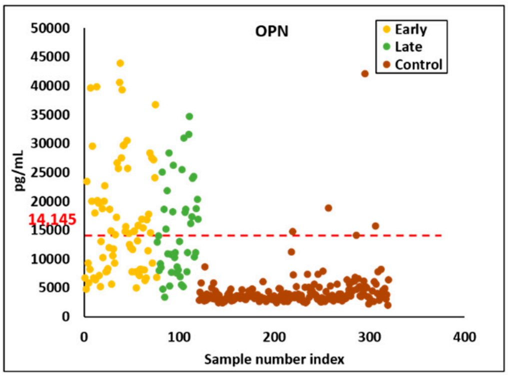

	Early	Late	Control
OPN (+)	38/76	20/44	4/200
%	50%	46%	2%

B

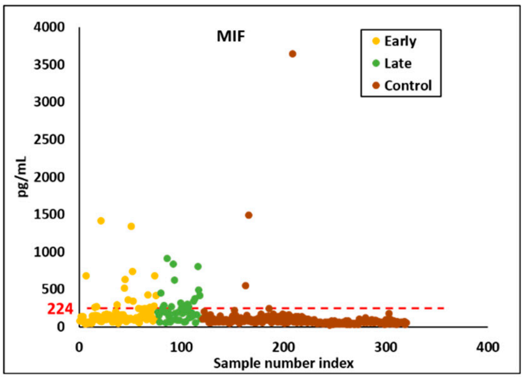

	Early	Late	Control
MIF (+)	16/76	17/44	4/200
%	21%	39%	2%

C

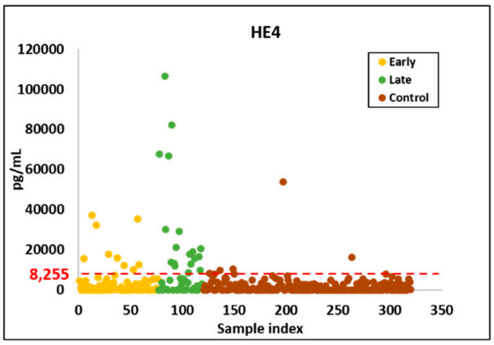

	Early	Late	Control
HE4(+)	9/76	18/44	4/200
%	12%	41%	2%

D

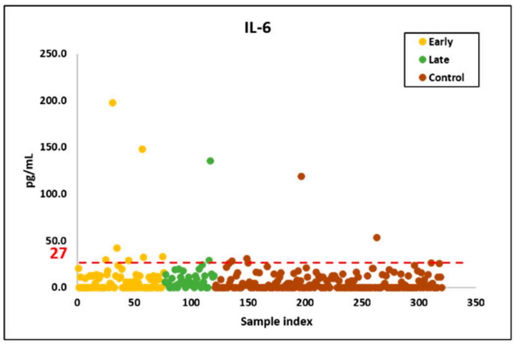

	Early	Late	Control
IL-6 (+)	7/76	2/44	4/200
%	9%	5%	2%

Figure S1. Serum levels of OPN, MIF, HE4 and IL-6 are elevated in the discovery set from ovarian cancer patients and healthy controls. (**A**) OPN antigen; (**B**) MIF antigen; (**C**) HE4 antigen and (**D**) IL-6 antigen. Each symbol represents the average of duplicate serum samples from individual early stage ovarian cancer cases (yellow), late stage ovarian cancer cases (green) or controls (red). The red dashed lines in each plot represent the cut-off value at 98% specificity for OPN, MIF HE4 and IL-6, and cut-off values are on the left side of one each graph.

A

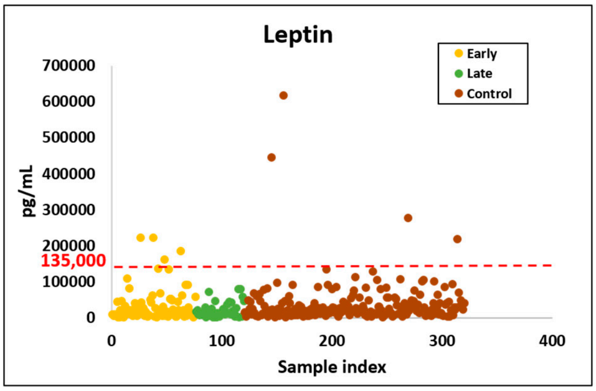

	Early	Late	Control
Leptin(+)	6/76	0/44	4/200
%	8%	0%	2%

B

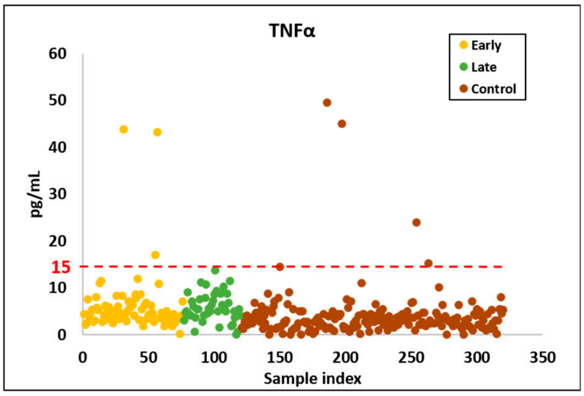

	Early	Late	Control
TNFα (+)	3/76	0/44	4/200
%	4%	0%	2%

C

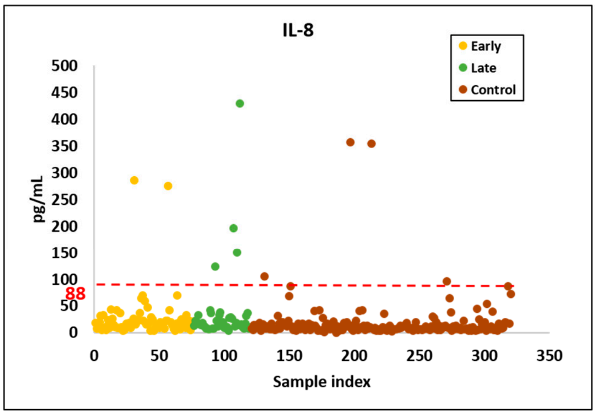

	Early	Late	Control
IL-8 (+)	2/76	4/44	4/200
%	3%	9%	2%

D

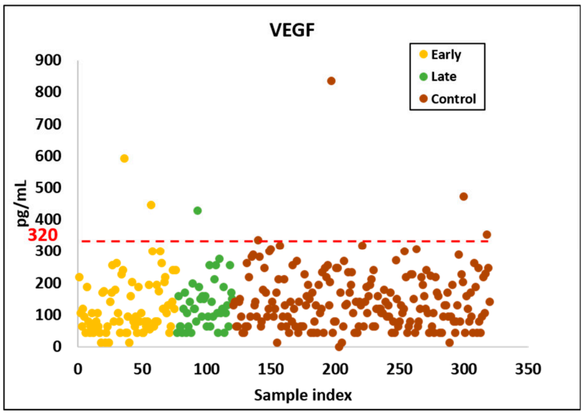

	Early	Late	Control
VEGF (+)	2/76	1/44	4/200
%	3%	2%	2%

Figure S2. Serum levels of Leptin, TNFα, IL-8 and VEGF are elevated in the discovery set from ovarian cancer patients and healthy controls. (**A**) Leptin antigen; (**B**) TNFα antigen; (**C**) IL-8 antigen and (**D**) VEGF antigen. Each symbol represents the average of duplicate serum samples from a individual early stage ovarian cancer cases (yellow), late stage ovarian cancer cases (green) or controls (red). The red dashed lines in each plot represent the cut-off value at 98% specificity for Leptin, TNFα, IL-8 and VEGF, and cut-off values are on the left side of one each graph.

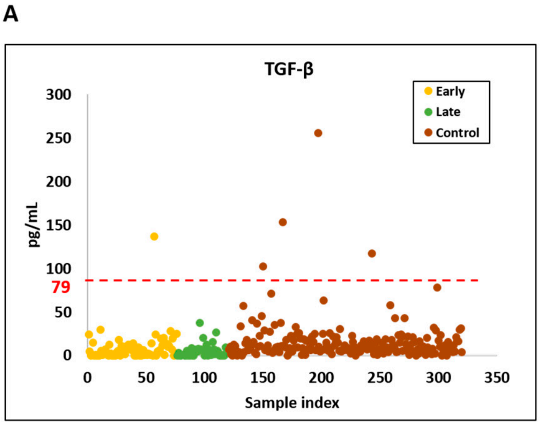

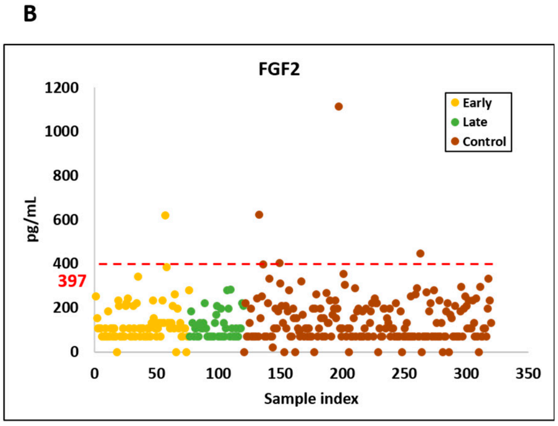

	Early	Late	Control
TGF-β (+)	1/76	0/44	4/200
%	1%	0%	2%

	Early	Late	Control
FGF2 (+)	1/76	0/44	4/200
%	1%	0%	2%

Figure S3. Serum levels of TGF-β, FGF2 are elevated in the discovery set from ovarian cancer patients and healthy controls. (**A**) TGF-β antigen; (**B**) FGF2antigen. Each symbol represents the average of duplicate serum samples from individual early stage ovarian cancer cases (yellow), late stage ovarian cancer cases (green) or controls (red). The red dashed lines in each plot represent the cut-off value at 98% specificity for Leptin, IL-6, TNFα and VEGF, and cut-off values are on the left side of one each graph.

A

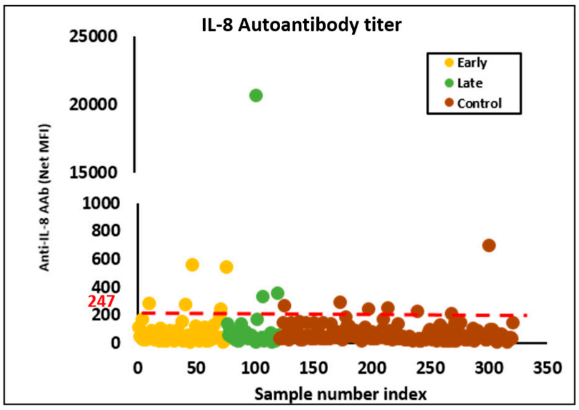

	Early stage	Late stage	Control
IL-8AAb (+)	5/76	3/44	5/200
%	7%	7%	3%

B

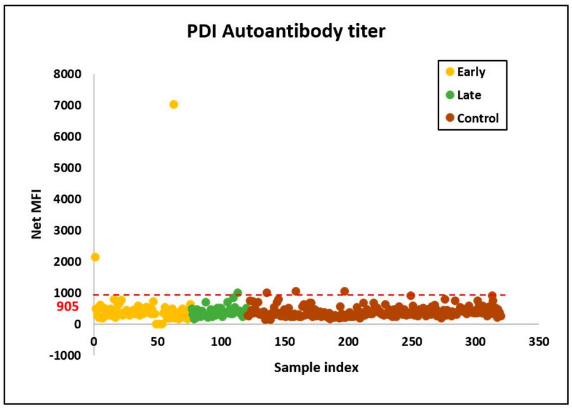

	Early stage	Late stage	Control
PDI(+)	2/76	1/44	4/200
%	2.6%	2.3%	2.0%

C

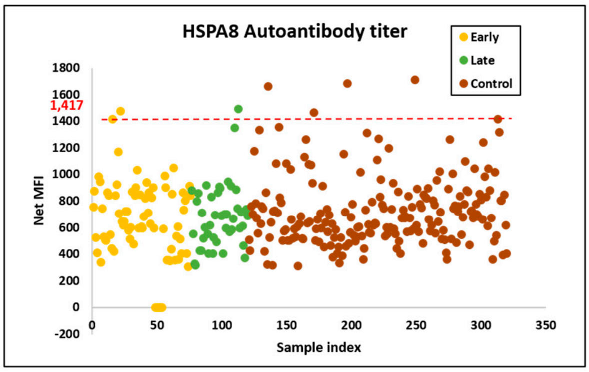

	Early stage	Late stage	Control
HSPA8(+)	2/76	1/44	4/200
%	2.6%	2.3%	2.0%

D

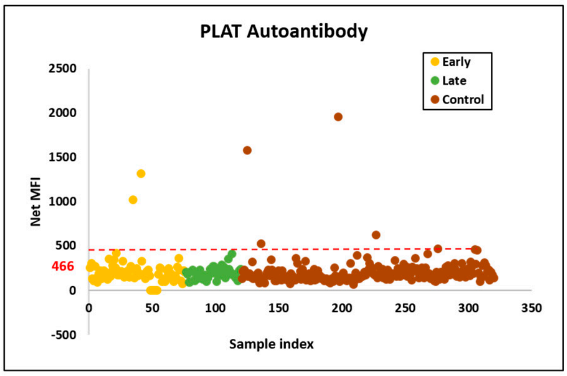

	Early stage	Late stage	Control
PLAT(+)	2/76	0/44	4/200
%	2.6%	0.0%	2.0%

Figure S4. Serum autoantibody titers (AAb) of IL-8, PDI, HSPA8 and PLAT are elevated in the discovery set from ovarian cancer patients and healthy controls. (**A**) IL-8 AAb; (**B**) PDI AAb; (**C**) HSPA8AAb and (**D**) PLAT AAb. Each symbol represents the average of duplicate serum samples from individual early stage ovarian cancer cases (yellow), late stage ovarian cancer cases (green) or controls (red). The red dashed lines in each plot represent the cut-off value at 98% specificity for IL-8, PDI, HSPA8 and PLAT AAbs, and cut-off values are on the left side of one each graph.

A

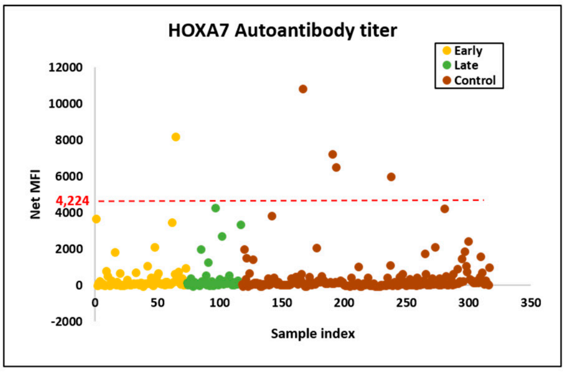

B

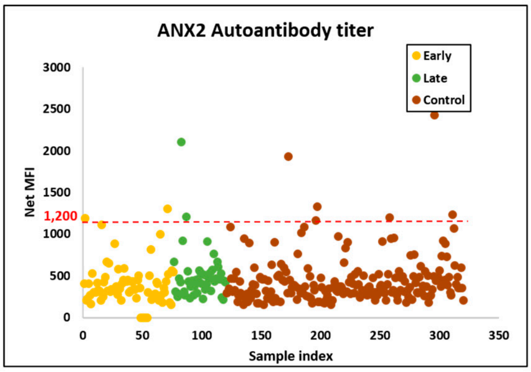

	Early stage	Late stage	Control
HOXA7(+)	1/73	1/44	4/200
%	1.4%	2.3%	2.0%

	Early stage	Late stage	Control
ANX2(+)	1/76	2/44	4/200
%	1.3%	4.5%	2.0%

C

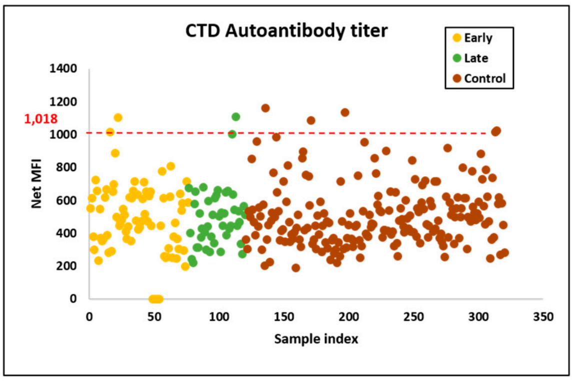

D

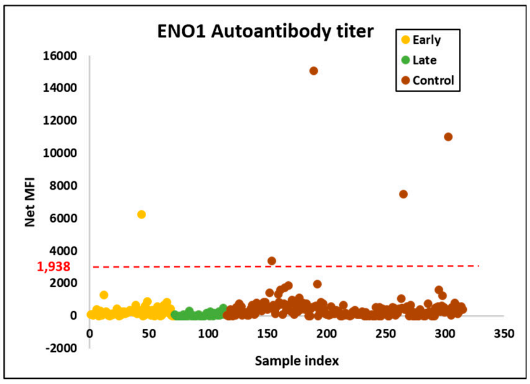

	Early stage	Late stage	Control
CTD(+)	1/76	1/44	4/200
%	1.3%	2.3%	2.0%

	Early stage	Late stage	Control
ENO1(+)	1/71	0/44	4/200
%	1.4%	0.0%	2.0%

Figure S5. Serum autoantibody titers (AAb) of HOXA7, ANX2, CTD and ENO1 are elevated in the discovery set from ovarian cancer patients and healthy controls. (**A**) HOXA7 AAb; (**B**) ANX2 AAb; (**C**) CTD AAb and (**D**) ENO1 AAb. Each symbol represents the average of duplicate serum samples from individual early stage ovarian cancer cases (yellow), late stage ovarian cancer cases (green) or controls (red). The red dashed lines in each plot represent the cut-off value at 98% specificity for HOXA7, ANX2, CTD and ENO1 AAbs, and cut-off values are on the left side of one each graph.

A

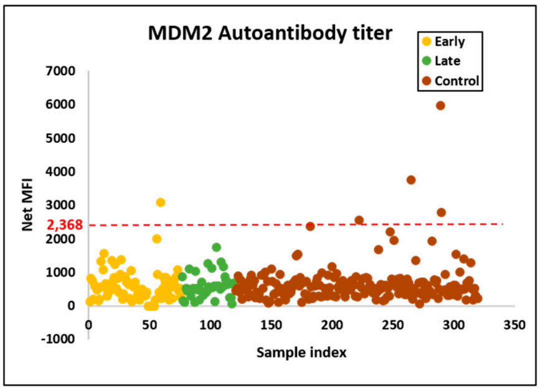

	Early stage	Late stage	Control
MDM2 (+)	1/76	0/44	4/200
%	1.3%	0.0%	2.0%

B

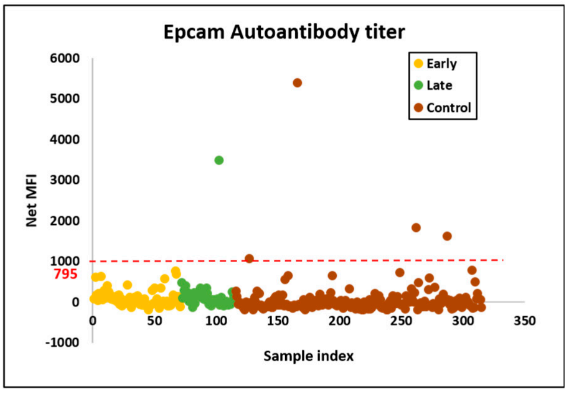

	Early stage	Late stage	Control
Epcam (+)	0/71	1/44	4/200
%	0.0%	2.3%	2.0%

C

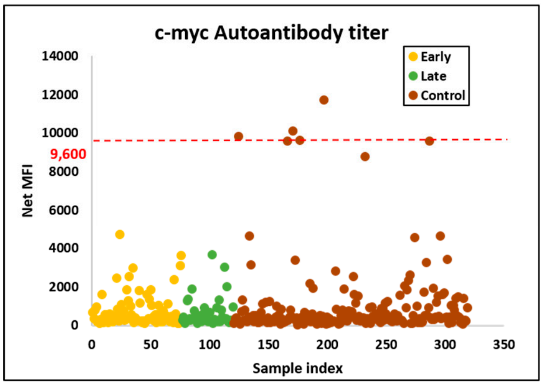

	Early stage	Late stage	Control
c-myc(+)	0/76	0/44	4/200
%	0.0%	0.0%	2.0%

D

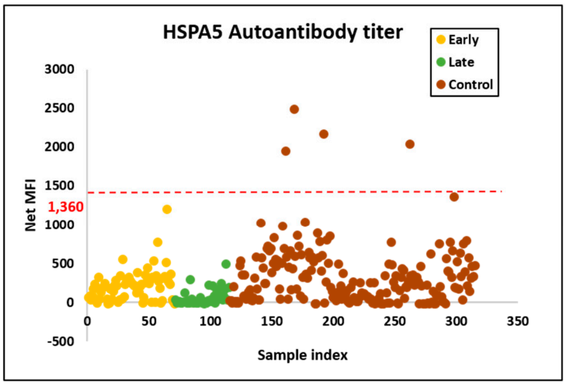

	Early stage	Late stage	Control
HSPA5(+)	0/76	0/44	4/200
%	0.0%	0.0%	2.0%

Figure S6. Serum autoantibody titers (AAb) of MDM2, Epcam, c-myc and HSPA5 are elevated in the discovery set from ovarian cancer patients and healthy controls. (**A**) MDM2 AAb; (**B**) Epcam AAb; (**C**) c-myc AAb and (**D**) HSPA5 AAb. Each symbol represents the average of duplicate serum samples from individual early stage ovarian cancer cases (yellow), late stage ovarian cancer cases (green) or controls (red). The red dashed lines in each plot represent the cut-off value at 98% specificity for MDM2, Epcam, c-myc and HSPA5 AAbs, and cut-off values are on the left side of one each graph.

Table S1. Screening results of 22 candidates in discovery sample set.

	Biomarkers	Sensitivity (%) at 98% SP	
		Early	Late
Antigen	Osteopontin (OPN)	50	46
	Macrophage migration inhibiting factor (MIF)	21	39
	Human Epididymis Protein (HE4)	12	41
	IL-6	9	5
	Leptin	8	0
	TNF-α	4	0
	IL-8	3	9
	VEGF	3	2
	TGF-α	1	0
	FGF2	1	0
Autoantibody	IL-8	7	7
	PDI	3	2
	HSPA8 (HSC70)	3	2
	PLAT	3	0
	HOXA7	1	2
	Annexin 2 (ANXA2)	1	5
	Cathepsin D	1	2
	Enolase 1 (ENO1)	1	0
	MDM2	1	0
	Epcam	0	2
	c-myc	0	0
	HSPA5 (GRP78)	0	0
